# Potential Therapies by Stem Cell-Derived Exosomes in CNS Diseases: Focusing on the Neurogenic Niche

**DOI:** 10.1155/2016/5736059

**Published:** 2016-04-19

**Authors:** Alejandro Luarte, Luis Federico Bátiz, Ursula Wyneken, Carlos Lafourcade

**Affiliations:** ^1^Laboratorio de Neurociencias, Facultad de Medicina, Universidad de Los Andes, Monseñor Alvaro del Portillo 12455, Las Condes, 7550000 Santiago, Chile; ^2^Center for Interdisciplinary Studies on the Nervous System (CISNe), Universidad Austral de Chile, Valdivia, Chile; ^3^Program for Cell Biology and Microscopy, Universidad Austral de Chile, Valdivia, Chile; ^4^Instituto de Anatomía, Histología y Patología, Facultad de Medicina, Universidad Austral de Chile, Valdivia, Chile

## Abstract

Neurodegenerative disorders are one of the leading causes of death and disability and one of the biggest burdens on health care systems. Novel approaches using various types of stem cells have been proposed to treat common neurodegenerative disorders such as Alzheimer's Disease, Parkinson's Disease, or stroke. Moreover, as the secretome of these cells appears to be of greater benefit compared to the cells themselves, the extracellular components responsible for its therapeutic benefit have been explored. Stem cells, as well as most cells, release extracellular vesicles such as exosomes, which are nanovesicles able to target specific cell types and thus to modify their function by delivering proteins, lipids, and nucleic acids. Exosomes have recently been tested* in vivo* and* in vitro* as therapeutic conveyors for the treatment of diseases. As such, they could be engineered to target specific populations of cells within the CNS. Considering the fact that many degenerative brain diseases have an impact on adult neurogenesis, we discuss how the modulation of the adult neurogenic niches may be a therapeutic target of stem cell-derived exosomes. These novel approaches should be examined in cellular and animal models to provide better, more effective, and specific therapeutic tools in the future.

## 1. Introduction

Highly prevalent CNS disorders that are associated with neurodegeneration include Parkinson's Disease (PD), Alzheimer's Disease (AD), Huntington Disease (HD), stroke, and epilepsy. The classification of neurodegenerative disorders is especially challenging, as different disorders may share similar clinical manifestations. Still, classifications are nowadays based on those clinical manifestations and/or the site of the brain that is affected: disorders affecting the basal ganglia in the forebrain affect movement, and these can be divided into hypokinetic (e.g., PD) or hyperkinetic (e.g., HD). An example of a disorder that involves the cerebral cortex that develops into dementia is AD, whereas an example of one involving the spinal cord is amyotrophic lateral sclerosis (ALS) [1]. A common trait for a considerable number of these disorders is, through disparate mechanisms, the accumulation of insoluble proteins, either extra- or intracellularly. AD is characterized by the aggregation of *β*-amyloid and the microtubule associated protein Tau; PD by the accumulation of the nerve terminal protein *α*-synuclein; ALS by deposition of phosphorylated TDP43 (a transcriptional repressor) and an enzyme that removes superoxide radicals, superoxide dismutase 1 (SOD1); and HD by the accumulation of the mutant protein huntingtin [2, 3]. Despite the wealth of knowledge that has been generated in the past decades, a reliable cure for neurodegenerative disorders remains elusive. There are many reasons with varied degrees of difficulty behind this inability to transform experimental information into successful medical treatments. The complexity of the central nervous system (CNS) and the multifactorial nature of these disorders are the most obvious challenges researchers have to tackle when attempting to predict the onset of a pathology and to ameliorate the burden of neurological disorders. In this review, we will describe evidence showing that stem cell-derived exosomes might be a new treatment for several CNS disorders considering a new interplay with neurogenic niches. Thus, we propose that engineered stem cell-derived exosomes targeted to the neurogenic niche are agents with therapeutic potential.

## 2. Management of CNS Disorders: Current State

Even when animal models are indispensable and have provided researchers with important and detailed information on the development and impact of neurological disorders, most models are still incapable of faithfully reproducing these disorders [4, 5], thus providing an incomplete tool to fully test the advantages of new drugs or treatments. Further complications arise when considering that many disorders progress slowly and, as mentioned before, that seemingly different disorders may be happening simultaneously. Furthermore, a disorder may express an important number of apparently disparate debilitating problems (Huntington Disease, e.g., is characterized not only by movement disorders but by a wide array of cognitive and behavioral disabilities [6]). Considering these adversities, a considerable amount of effort has been placed not only on treating a specific disorder, but also on discovering biomarkers that may facilitate predicting or recognizing neurodegenerative pathologies during the early stages of its development, where treatments are usually most effective.

The currently used methods to diagnose neurodegenerative disorders ante-mortem may range from neuropsychological assessments (e.g., cognitive tests to evaluate memory loss in patients suffering from Alzheimer disease (AD) [7]) to neuroimaging. These methods are not always reliable, due in part to the difficulty imposed when trying to differentiate them from other disorders that share common features. For instance, diagnosis of Parkinson's Disease (PD) can be confounded by other diseases that present clinical syndromes of parkinsonism, and a study applying recent criteria [8] to diagnose patients with AD was highly successful (95%) when considering nondemented patients, but this success rate decreased almost by half when considering a population of patients with other types of dementia [9]. These difficulties are being tackled, and they can be divided for organizational purposes in attempts to discover reliable biomarkers on the one hand and endeavors to find appropriate therapies on the other hand.

### 2.1. Common Biomarkers for CNS Diseases

Currently used biomarkers rely heavily on imaging technologies, which can be used to study from whole brain structures to protein aggregates. Magnetic resonance imaging (MRI) has been used, for example, to associate changes in ventricular volume with cortical senile plaques (SP) and neurofibrillary tangles (NFT), common features of AD, thus being proposed as a diagnostic strategy [10]. Nevertheless, it may be difficult to separate the changes observed in AD patients from the changes observed in those suffering other types of dementia or even the elderly people [11, 12]. Diffusion MRI has proven successful in identifying PD patients from those suffering from parkinsonism [13], and diffusion-weighed MRI and computer tomography (CT) are the most common ways of detecting early cases of acute stroke [14, 15]. Despite the sometimes prohibitive costs, positron emission tomography (PET) is a promising option for early diagnostic of a disorder, as well as a powerful tool to determine the success of a treatment over time, although the injection of radioactive tracers in the blood makes PET a slightly more invasive technique. Newly developed tracers for PET have expanded the possibilities of this technique, allowing it to detect not only the activity of the brain through blood flow and glucose consumption but also parameters that may signal the development of a neurological disorder [16]. Examples of these tracers include ^18^F-DOPA, which has been used to diagnose PD, and compounds that bind to amyloid plaques, such as ^18^F-florbetapir, which presents a higher uptake in AD patients than controls [17] and has been recently approved by the FDA [18]. Magnetic resonance spectroscopy has also been used to diagnose early stages of PD, showing an interesting potential when considering the noninvasiveness of this technology and the lower associated costs [19].

### 2.2. Searching for New Noninvasive Biomarkers for CNS Diseases: Relevance of Exosomes and MicroRNAs

Body fluids are also promising sources of molecular biomarkers, which can be divided into three categories: molecules (e.g., 8-hydroxydeoxyguanosine, a byproduct of DNA oxidation that is found at higher levels in the urine of PD patients), proteins (e.g., protein aggregates), and RNAs (e.g., noncoding microRNAs; see below) [20]. The advantage of biomarkers obtained from body fluids (i.e., cerebrospinal fluid, CSF, blood, plasma, serum, saliva, and urine) is the possibility of searching for a large number of molecules at once, for example, by the use of proteomics or genomics, at earlier stages than those exposed by imaging. Efforts are being made to measure the higher levels of glial fibrillary acidic protein (GFAP) in blood, for example, as an early marker of traumatic brain injuries and stroke, as well as a way to follow up on a specific treatment for these patients [21]. Having a biomarker that can be accessed from a body fluid has the added advantage of not relying on more expensive technology (such as imaging equipment) and, in some instances (e.g., saliva and urine), of avoiding invasive methods altogether. The main disadvantage of obtaining biomarkers from body fluids is the low levels of molecules and the heterogeneity of these, as such samples arise from a wide number of tissues. Thus, to circumvent this difficulty and to improve the specificity of the biomarker, small circulating extracellular vesicles termed exosomes have gathered the interest of biomedical researches. These extracellular nanovesicles can be isolated from all bodily fluids, and they carry a complex cargo consisting of various types of RNA (e.g., ribosomal RNAs, long noncoding RNAs, and microRNAs), proteins, lipids, and DNA that in part depends on the tissue of origin and its “health or disease” state [22–24]. Catalytically active enzymes like PTEN, as well as bioactive lipids such as prostaglandins, can be transfered by exosomes to target cells [25, 26].

Exosomes carry a set of common proteins considered as “exosome markers,” most of them related to their biogenesis [22]. In addition, they carry molecules that reflect their cellular origin, for example, membrane and intraluminal proteins. In the case of transmembrane proteins, they can be used to immunoisolate exosomes of a specific cellular origin, separating them from other exosomes and thus improving the sensitivity of exosomes as biomarkers [27]. In line with that, neurodegenerative disorders are characterized by exosomes that carry the misfolded protein type found in these disorders (e.g., *β*-amyloid in AD and *α*-synuclein in PD) [28]. Of all the molecules carried by exosomes, microRNAs (miRNAs) are the ones that have gathered the most interest in the last years. These short (~22 nucleotides long) noncoding RNAs are considered as “master regulators” of translation; one miRNA may repress translation of several (even a hundred) mRNAs. One of the aspects that make miRNAs per se promising biomarkers is that they can be found in body fluids that are easily accessible, such as plasma, where they appear to be transported by lipoproteins and exosomes or bound to the protein argonaute-2, a key component of the silencing complex mediated by miRNAs [29]. Exosomes provide an enriched source of miRNAs for biomarker profiling [30], and sets of miRNAs obtained from exosomes circulating in the blood have been proposed as biomarkers for cancer diagnostics [31, 32]. More recently, miRNAs present in blood-derived exosomes have been linked to specific neurodegenerative disorders, such as AD, PD, and brain injury [33, 34].

In this context, the possibility of extracting high quality miRNAs and profiling them using well-established methods has also contributed to making them a favored area of study in the search for biomarkers [35]. Researchers have attempted to profile the miRNA identity from body fluids of patients with the most common neurodegenerative disorders, and even when the number of miRNAs associated with neurodegenerative disorders is continually increasing, there are important difficulties that need to be considered if some of these molecules are going to be proposed as reliable biomarkers. The methodologies used for these profiles are not consistent between laboratories, and the sample sizes are usually small; thus, validating the miRNAs associated with neurological disorders has proven difficult [36]. Therefore, although there are increasing reports on the application of exosome-derived miRNAs as biomarkers for various diseases, it is still an ongoing process with a considerable degree of variability and efforts are constantly made to increase specificity and sensitivity [37].

## 3. Need for New Therapies in Debilitating CNS Diseases

Perhaps the most striking advance in the treatment of a neurodegenerative disorder was the discovery made half a century ago that administration of L-dopa, a precursor of the neurotransmitter L-dopamine, improved many of the symptoms associated with PD, in which dopamine neurons in the substantia nigra degenerate. Thus, this treatment merely improves symptoms by elevating levels of the neurotransmitter, but it does not replace or improve survival of degenerating neurons. In spite of this shortcoming, this drug still remains the main line of treatment for PD patients, though it may present side effects such as dyskinesias, and it is unable to alleviate the nondopaminergic symptoms (e.g., dementia and psychiatric disorders) that become more prevalent as the disease progresses [38, 39]. The success of L-dopa has not been replicated when trying to modify the neurotransmitter milieu affecting other neurological disorders associated with degeneration of specific neuronal populations; gamma-aminobutyric acid (GABA) agonists were unable to improve patients suffering from Huntington's disease, and cholinesterase inhibitors have not been successful in significantly improving the cognitive impairments observed in patients of AD [40]. This has led to exploring other alternatives, for example, related to more efficient delivery of drugs across the blood-brain barrier with the use of nanotechnology [41] and to the use of vaccines against protein aggregates characteristic of AD (tau or *β*-amyloid [42]).

## 4. Stem Cells as a New Treatment for CNS Diseases

Among the innovative therapeutic strategies, the use of stem cells has gained particular attention. These cells are capable of self-renewal and can be classified according to their capacity to form a specific tissue or cell lineage: totipotent cells can form all the cells of the organism, pluripotent cells can form the tissues from the three germinal layers (i.e., endoderm, ectoderm, and mesoderm), and multipotent cells can give rise to a certain lineage of cells. According to their origin, they can be divided into embryonic or adult (i.e., postnatal), and in both cases there are advantages and disadvantages associated with their use in the clinic. The main advantage of embryonic stem cells is their capacity to generate a vast number of cell types. The main disadvantages of these cells are the ethical concerns raised by the use of embryos and the likelihood of triggering an undesired immune response or the formation of tumors. Adult stem cells have a reduced potential compared to their embryonic counterpart, but since they can be obtained from the same patient, the chances of an immune response are significantly lower. The risk of tumor formation when using these cells is also lower, and the ethical controversies are avoided altogether [43–45]. A remarkable accomplishment was the reprogramming of adult somatic cells to pluripotent ones (induced pluripotent stem cells, iPSCs) by transfection of specific transcription factors [46, 47], therefore making them more similar to embryonic stem cells but without the degree of immunoreactivity and the ethical controversies of the latter. These cells are currently used to model a wide variety of diseases, from muscular to neurological disorders that have an elusive solution. The possibility that these cells lead to tumorigenic process due to incomplete reprogramming or the inhibition of tumor suppressor genes during the reprogramming stages has so far hindered their use in the clinic [48].

Mesenchymal stem cells (MSCs) are multipotent progenitors, with self-renewal capacity, that confer neuroprotection and can be isolated from umbilical cord (UC), bone marrow (BM), adipose tissue, and even menstrual fluids, among other sources [49]. MSCs have several features that make them useful for CNS disease treatment; for instance, they can be isolated by almost noninvasive procedure, being easily cultured and expanded (and thus suitable for molecular engineering). MSCs also have low immunogenicity and tumorigenic potential, their use has no ethical constraints [50], and several works have confirmed their healing abilities on the CNS. For example, MSCs obtained from the bone marrow improved behavioral outcomes in a rat model of PD and decreased depressive-like behavior by promoting neuronal growth and survival [51, 52]. Moreover, BDNF-producing human MSCs transplanted in the brain of a rodent PD model were able to integrate successfully and deliver trophic factors [53]. MSCs from umbilical cord enhance brain angiogenesis after stroke [54] and MSCs from adipose tissue increase the number of motor neurons and motor outcomes in a mouse model of ALS [55].

Multipotent stem cells can also be found in neurogenic niches of the adult CNS, continuously giving rise to neurons or glial cells. This neurogenic process is severely affected in several CNS diseases [56, 57]; thus, the idea of regulating the neurogenic niche in neurological disorders has recently emerged [23, 50, 56].

## 5. Importance of Neurogenic Niches in CNS Disorders: A Potential Therapeutic Target

In the mammalian brain, there are defined regions termed neurogenic niches, areas with the proper environment that are able to support and modulate neurogenesis during adulthood [58]. The first validated and most studied neurogenic niches of the brain are the subventricular zone (SVZ) of the lateral ventricles and the subgranular zone (SGZ) of the dentate gyrus in the hippocampus [59]. Nevertheless, other brain regions have been proposed as having putative neurogenic niches (e.g., substantia nigra, cerebellum, and amygdala) though the extent at which this happens* in vivo* and in humans remains controversial for some of them. Neurogenesis has been shown to occur in the spinal cord of primates after injury [60], and recent studies have shown that adult neurogenesis is active in the hippocampus [61] and in the striatum [62, 63].

These findings raise the question as to whether such processes can be manipulated for therapeutic purposes. A number of experiments have already shown the impact that some disorders have on these niches and their role in improving pathological conditions. Animal models of chronic stress show a reduction in the levels of hippocampal neurogenesis, and some of the beneficial actions brought upon by antidepressants have been shown to involve modulation of the neurogenic niche [64–66].

**Figure 1:**
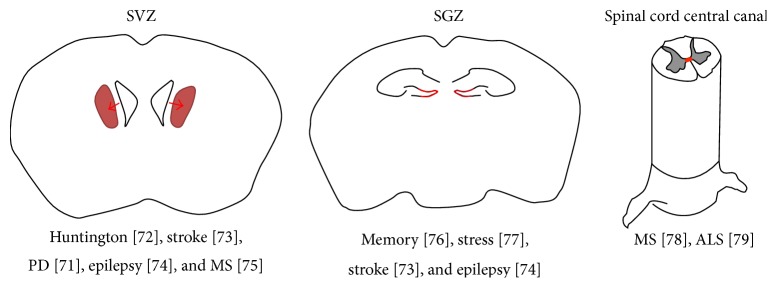
Animal models of CNS diseases (and memory processing) potentially linked to neurogenic niche alterations. Schematic representation of brain coronal slices and transverse section of spinal cord. Neurogenic niches are indicated in red lines and arrows. SVZ corresponds to subventricular zone; SGZ corresponds to subgranular zone; spinal cord central canal niche is also depicted. The reference number for each pathology associated with the neurogenic niche is indicated. References: PD [71]; Huntington [72]; stroke [73]; epilepsy (SVZ) [74]; MS (SVZ) [75]; memory [76]; stress [77]; stroke (SGZ) [73]; epilepsy (SGZ) [74]; MS (spinal cord central canal) [78]; ALS [79].

In postmortem brain tissue of humans with PD, there is a reduction of proliferating cells in the subependymal zone (SVZ) and the SGZ, and similar results have been observed in animal models of PD. Proteins like *α*-synuclein may be one of the molecules playing an important role in these mechanisms, as their accumulation disrupts adult neurogenesis [67, 68]. The impact of AD in adult neurogenesis is more elusive, as both the increase and reduction of neurogenic markers have been reported on the hippocampus of humans, discrepancies that may be partially explained by the severity of the disorder at the time the samples were gathered, for example, tissue obtained from the early stages versus samples collected from the later and more severe manifestations of the pathology. Similarly, animal models show disparate results that may also depend on the model being used, age, and strain of the animals, among others [57]. Neurogenesis is enhanced after a stroke episode in humans and in animal models, and recovery after a stroke in animal models is facilitated or diminished whether neurogenesis is enhanced or prevented, respectively [69, 70]. Human striatal neurogenesis is gradually reduced in HD as well [62].  Figure 1 shows a summary of several animal models of CNS diseases (and memory processing) related to the proper functioning of neurogenic niches.

MSCs may act by regulating neurogenic niche function as has been already shown in the SGZ of the hippocampus, where implantation of these cells in the DG increased the proliferation of endogenous NSCs as well as their differentiation into neurons [80]. It was also shown that cisterna magna injections of human UC-MSCs activated endogenous hippocampal neurogenesis and significantly reduced A*β*42 levels [81]. In fact, there seems to be enough evidence to propose that the interplay between MSCs and the different neurogenic niches could be a key factor in the intervention of several CNS pathologies [50]. Nevertheless, the regenerative properties of MSCs when injected are probably indirect, as only a small proportion of the cells transplanted reach their target zone [82]. Coherently, administration of MSCs after brain injury induced recovery with low MSCs engraftment in the ischemic zone [83]. Therefore, therapeutic effects are thought to arise from the release of extracellular factors (membrane-bound and soluble); among them, extracellular vesicles like exosomes have gained much attention. Next, we will provide a detailed description of the nature of these vesicles in order to understand their potential as therapeutic tools that modulate neurogenic niche function.

## 6. Extracellular Vesicles and Exosomes

Several kinds of extracellular vesicles (EVs) have been described nearly 30 years ago; among them are exosomes, characterized by a nanosize of 30–100 nm; apoptotic bodies of around 1 *μ*m; and ectosomes of 100 nm–1 *μ*m, a concept in which microvesicles, microparticles, and shedding vesicles have been included [84–86]. While exosomes originate from the endocytic route, the rest of the vesicles emerge directly from plasma membrane. The molecular components that give rise to exosomes are highly conserved among most of eukaryotic organisms and there is evidence showing that virtually all kinds of cells and extracellular fluids (even in protozoa) contain exosome-like EVs [87, 88]. It is important to have in mind that the precise distinction from other kinds of EVs is to some extent difficult [89]. EVs enriched in exosomes are harvested from extracellular fluids after several centrifugation steps to get rid of floating cells and cellular debris followed by one or two ultracentrifugation steps that end up with the recovery of a pellet obtained at 100,000 ×g (or more) for at least one hour [90]. In the present review, we will use an operational definition of the EV fraction we are calling exosomes, that is, pellets obtained from extracellular fluids (depleted of cells and debris) that were collected after 100,000 ×g ultracentrifugations for at least one hour. Nevertheless, all EV types have common properties that allow them to mediate intercellular interactions, eliciting several kinds of cellular responses in target cells that we will further discuss.

### 6.1. Exosome Biogenesis and Content

The concept of exosomes as extracellular vesicles was first settled with the description of multivesicular bodies' (MVBs) fusion with the plasma membrane during the maturation of reticulocytes to red blood cells, where intraluminal vesicles (ILVs) are released to the extracellular space [91–94]. These structures became notorious because of their novel location (outside the cell) and their peculiar topologic organization in which the lumen is equivalent to the cytosolic portion of the cell and its membrane has the same orientation of the plasma membrane. Much effort has been done to elucidate the mechanism of MVBs biogenesis and the sorting of cargo into ILVs [22, 89, 95, 96]. Two main ways for destining cellular components to ILVs have been described: the ESCRT (endosomal sorting complex required for transport) dependent and ESCRT independent mechanisms. The ESCRT machinery is composed of four complexes (ESCRT-0, ESCRT-I, ESCRT-II, and ESCRT-III) that sequester ubiquitinated membrane proteins into an endosomal microdomain and induce an invagination that generates ILVs formation harboring this cargo. Sorting mechanisms of soluble proteins into exosomes have been related to microautophagy [97] or physical interactions with sorted transmembrane proteins, but it is still a less explored field. On the other hand, ESCRT independent incorporation of cellular components to exosomes is mediated by ceramide induced ILVs budding [98, 99]. Other proteins are sorted by variations of the canonical ESCRT dependent model [100, 101]. In relation to the incorporation of nucleic acids into ILVs, some miRNAs harboring the GGAG motif in the 3′ portion bind to sumoylated proteins such as hnRNPA2B1 prior to its destination into ILVs [102]. Another destination motif for miRNA to exosomes was found to be dependent on uridylation also at 3′ end of the nucleic acid [103].

Exosomes contain proteins related to several cellular functions such as vesicular transport (Rab GTPases, annexins, and flotillins), heat shock (HCP/HSP 70 and 90), MVBs biogenesis (Alix and TSG 101), integrins and tetraspanins (CD63, CD9, CD81, and CD82) [104, 105], cytoskeletal proteins (actin, syntenin, and moesin), signal transduction proteins (kinase proteins), and metabolic enzymes (GAPDH, LDHA, PGK1, aldolase, and PKM) [106]. Exosome markers are typically enriched in MVBs. Thus, some markers are cytosolic proteins like HSP70, which mediates microautophagy of cytosolic proteins to MVBs [97], and proteins related to exosome biogenesis such as programmed cell death 6 interacting protein (PDCD6IP), also known as ALIX, and tumor susceptibility gene 101 protein (TSG101). Besides, membrane associated proteins like LAMP-3 (or CD63, enriched in late endosomes and lysosomes) [107], CD81, MHCII (restricted to specific cell types [108]), and CD9 [90] as well as lipid raft enriched proteins such as flotillin-1 [109] and flotillin-2 [110] are considered exosome markers. In addition, there are proteins that have been shown to be absent in different exosome preparations, such as proteins from the endoplasmic reticulum, for example, Gp96, calnexin, and the Golgi apparatus, for example, GM130, and from mitochondria, for example, cytochrome C [90, 111]. In addition, exosomal membranes harbor a characteristic lipid profile that resembles a lipid raft composition, containing cholesterol, ceramide, sphingomyelin, and phosphatidyl-serine [112].

### 6.2. Mechanisms of Interaction of Exosomes with Target Cells

A huge volume of data shows that exosomes transfer several kinds of functional biomolecules that gate a wide spectrum of changes on cellular processes [25, 113–117]. Several uptake mechanisms have been proposed for internalizing a vesicle into a cell, most of which are mediated by the endocytic route such as clathrin mediated endocytosis [118], phagocytosis [119], lipid rafts mediated internalization [120], and macropinocytosis [121] and also by direct fusion with the plasma membrane (although this is supposed to be a minor contribution [122] and also technically difficult to prove [113]). Regardless of the mechanism, before vesicles enter the cell, they may dock plasma membrane components, among which we can find integral transmembrane proteins such as CD81 or CD9 (which are also exosome markers), integrins like av (CD51) and b3 (CD61), and extracellular matrix components such as heparan sulfate proteoglycans (HSPGs) [123]. After delivery of exosomes into endocytic compartments, low pH would favor vesicle membrane fusion with cell membranes [117]; thus, most exosomal lipids would reach the plasma membrane indirectly as they come from the endocytic route. All this explains lipids and transmembrane associated proteins transfer, but what about functional delivery of miRNAs?

It has been shown that MVBs tend to be in close association with the RNA-induced silencing complex or RISC, a multiprotein complex, which incorporates miRNA and mediates the translational repression of a given target or mRNA degradation [124]. Although still not proven, endocytosed exosomes could behave as ILVs that fuse with MVBs internal surrounding membrane (called back-fusion [113]) that would allow RNAs, in the lumen of ILVs, to find the cytosolic RISC complex and be functional. The rest of the lumenal content should be also delivered this way to the cytosol.

Exosomes may elicit cellular responses by ligand mediated transductional pathways. Almost a decade after the first description of exosomes, Raposo et al. [125] described exosomes as carriers of major histocompatibility complexes (like MHCII) loaded with antigenic peptides that were able to help B lymphocytes during the immune priming of T cells. Furthermore, the same group showed that dendritic cell-derived exosomes, harboring MHCII loaded with tumoral epitopes, could function as a brand new oncogenic treatment [126]. Since then, several works have tried to use exosomes as carriers of molecular, and even pharmacological, factors.

## 7. Therapeutic Potential of Stem Cell-Derived Exosomes: Focusing on the Neurogenic Niche

One of the key limits for a noninvasive systemic therapy of CNS disorders is the fact that several substances are not able to cross the blood-brain barrier (BBB). This is a multicellular interface composed, roughly, of pericytes, astrocyte's end feet, and an astrocyte induced modified epithelium that becomes paracellularly impermeable to certain molecules (e.g., drugs) and to most of the cells of the bloodstream [127]. In relation to stem cell therapy, there is evidence that systemically injected MSCs are able to cross the BBB and reach the “damaged zone” of the brain, although this is to some extent controversial because the integrity of the BBB can be compromised by inflammatory conditions [128].

Regarding new potential therapies for CNS disorders, one of the most outstanding results in the field is the fact that systemically injected exosomes are able to cross the BBB and achieve the brain parenchyma. For instance, it was shown that neurons can be specifically targeted to receive a functional siRNA using previously transfected and engineered exosomes [129]. The same group was able to show that systemic delivery of targeted exosomes containing a siRNA against *α*-synuclein reduced the mRNA and protein levels of *α*-synuclein in the brain [130]. In a similar approach to circumvent the BBB constraints, it has been shown that intranasally injected exosomes are able to deliver curcumin to microglia in the brain parenchyma, inducing a clear recovery in an animal model of multiple sclerosis termed experimental autoimmune encephalomyelitis [131]. The same administration pathway was used by this group to deliver miR-17 in nanovectors to inhibit brain tumor progression [132]. With respect to a PD model, it was found that macrophages-derived exosomes made to carry the enzyme catalase are able to reach brain parenchyma (also by intranasal administration) and induce neuroprotective changes in mice [133].

With regard to stem cell-based therapy, systemic administration of MSCs exosomes can improve some of the neurological conditions observed in animal models of stroke, and certain neuronal properties can be enhanced or reduced by upregulating or lowering the levels of specific miRNA in MSCs [134]. For example, bone marrow mesenchymal stem cells (BM-MSCs) treated with ischemic brain extract produced exosomes with neuroprotective effects in a stroke model in rats, inducing functional recovery mediated by transfer of miR-133b in these vesicles [135]. Similarly, Doeppner et al. found that systemically injected human BM-MSC-derived exosomes were able to improve angiogenesis and neurogenesis in mice. Interestingly, they found similar results with direct use of parental BM-MSCs [136]. With these recent results, several questions arise.

### 7.1. What Are the Advantages of Using Stem Cell-Derived Exosomes instead of Parent Stem Cells for Therapeutic Purposes?

Exosomes may provide a way to increase the possibilities of a cell to reach many other places in the body and, due to their small size, they expand the “interacting surface of a cell” in relation to its volume [137]. On that virtue, exosomes may increase the surface/volumen ratio and amplify ligand gated signaling pathways and the transfer of biomolecules from stem cells to target tissues.

There are several considerations about the advantages/risks of exosomes versus stem cell therapy, some of them already discussed elsewhere [138]. Among the risks of cell therapy with stem cells are negative tumor modulation, malignant transformation, and obstruction of small vessels. With regard to the cell therapy advantages, we might find the continuous release of exosomes (cell is alive), soluble factors, and the potential differentiation and replacement of damaged cells [138]. The advantages of exosome based therapy are low immunogenicity [139], no vascular obstructive effect [138], permeability through BBB [131], and the potential to develop large scale cellular factories of engineered therapeutic vesicles [86].

Following the idea that stem cell therapies are usually safe, it has been argued that the risk of stem cell-derived exosome (harboring the same components) mediated therapy is expected to be low [140]. Nevertheless, exosomes have specific physicochemical properties and a molecular signature that demands us to be cautious. For example, parental exosome producing cells may have very low or undetectable levels of the abnormally folded prion protein (PrP) scrapie (PrPsc) on its surface, but as previously mentioned, the smaller size of exosomes increases the surface/volumen ratio compared with the same amount of cells. In fact, it has been shown that exosomes released from infected cells containing PrP and PrPsc are infectious [141]. Thus, although it seems unlikely, stem cell-derived exosomes may encompass toxic features in some conditions that need to be assessed in depth in order to avoid them. Recently, several companies are researching the therapeutic use of EVs in regenerative medicine. Some cautions about the potential oncogenic features of EVs have been thoroughly discussed in the literature [142], and some articles have highlighted the main concerns that EV-based therapies should accomplish [140].

### 7.2. Could We Specify Key Components of Stem Cell-Derived Exosomes That Are Responsible for Their Therapeutic Effects?

Each of the exosomal components may provide a peculiar interacting mechanism. Recently, Katsuda and Oshiya discussed the contribution of RNAs and proteins as mediators of the therapeutic effect of mesenchymal stem cell-derived EVs [142]. This analysis suggests that most of the functional effects of exosomes were explained by the presence of RNAs or specific known miRNAs, instead of proteins. Although that was not an exhaustive search, it may reflect the fact that RNAs and miRNAs are the most relevant cargoes in exosomes in terms of the ability of a small number of molecules to influence several proteins/enzymes from one or more cellular pathways on target cells [143]. Perhaps one of the most compelling evidences on the importance of miRNA exosomal content is a recent result by Collino et al. [144], showing that the therapeutic effect of mesenchymal stromal cell-derived exosomes on acute kidney injury was abolished when exosomes were released by cells depleted of Drosha protein (producing a total downregulation of miRNAs).

Taking all of the above into consideration, previously mentioned evidence suggests that miRNAs are important components of the signaling mechanisms mediated by exosomes, as several of the functional effects can be reproduced by a specific miRNA or be reverted with its blockade/inhibition [134, 145]. In consequence, in the search for a therapeutic use of exosomes, their miRNA content should be a fundamental factor, if not the main, to be considered.

### 7.3. What Are the Advantages of Stem-Cells Exosomes Compared to Those Derived from Adult Cells?

The lack of clinical tests and rigorous comparisons makes it difficult to assess the advantage of using exosomes derived from adult cells compared to those obtained from stem cells. However, researchers have speculated that stem cell-derived extracellular vesicles, including exosomes, may potentially transmit some of the unique stem cell properties to other stem cells, facilitating stemness maintenance, differentiation, self-renewal, and repair [146–148] and that these properties appear to be independent of the tissue from where the stem cells were obtained [149]. Thus, stem cell-derived exosomes may recapitulate several features of their cells of origin and may facilitate the horizontal transfer of information that supports stem cell biology. In addition, stem cell-derived exosomes play a key role in the induction of reparative programs within injured tissues. Exosomes released from MSCs are a source of regeneration in several pathological environments and tissues such as myocardial infarction [149–151], drug-induced liver injury [152], endotoxin-induced acute lung injury [54, 153], and traumatic brain injury [154]. In the latter, exosomes derived from MSCs improve functional recovery after traumatic brain injury by promoting endogenous angiogenesis and neurogenesis and by reducing neuroinflammation [154].

This makes stem cell-derived exosomes particularly attractive compared to adult cell-derived exosomes, but more research will be needed to analyze the benefits and disadvantages of one over the other.

## 8. Engineering Exosomes to Target Them into the Neurogenic Niche

Exosomes have been engineered in several ways. Most of the modifications are aimed at docking the vesicle with the target cell using specific ligand/receptor binding strategies to facilitate endocytosis. For example, Ohno et al. used exosomes harboring a GE11 peptide fused to the platelet derived growth factor (PDGF) receptor transmembrane domain. The GE11 peptide has high affinity for the epidermal growth factor receptor (EGFR), a protein enriched on several human tumors of epithelial origin. Strikingly, GE11-positive exosomes that contained the miRNA let-7 were able to inhibit tumor development* in vivo* [155]. Another interesting experiment was done using modified EVs expressing the neuron-specific rabies viral glycoprotein (RVG) peptide on the membrane surface to deliver the siRNA targeting the opioid receptor mu into the brain. This EV treatment was shown to serve as a potential therapy for morphine addiction [156]. In this case, the RVG peptide was fused to LAMP2b, a protein that is highly expressed in exosomes, using a very similar approach to Alvarez-Erviti.

Though speculative, one might target the neurogenic niche in the CNS in order to increase differentiation of a specific cell type or region. For example, the subgranular zone in the hippocampus, related to mood disorders, could be reached by stem cell-derived exosomes to improve neurogenesis. Therefore, assessing specific molecular features of the stem cell niche might help improve exosomal targeting. Although attempts in that line have been undertaken [157], there is still insufficient information in the field. Nevertheless, we are including a brief proposal of molecules that might function to specifically target exosomes to the niche. Once a specific molecular target for delivery has been identified, the next step is to construct a recombinant protein fusing a mimetic peptide (able to bind target proteins) with the extracellular domain of a highly expressed exosome marker such as LAMP2, CD63, or flotillin-1.

Although the knowledge of specific markers for neurogenic niches is scarce, there are few enriched proteins exposing an extracellular domain that would be able to dock exosomes to certain cells. For example, it has been shown that the neurogenic niche expresses the gap junction proteins connexin 43 and connexin 26. While connexin 43 is also enriched in astrocytes [158], connexin 26 has been shown to be enriched in the neurogenic niche associated with the subependymal layer (SVZ) [159]. This enrichment is useful as it has been shown that Cx 43 mediates exosome docking and internalization with target cells [160]. Thus, the extracellular domain of a tetraspanin (e.g., CD63) could be fused with a mimetic peptide similar to others that are known to bind connexins [161, 162] or even to the small domain of Cx26 that retains the ability to interact with cellular hemichannels. Another potential source to achieve specificity is to use the extracellular protein tenascin C. Tenascin C is highly enriched in the SVZ from embryonic and adult mice [163, 164]. This protein is mainly expressed in astrocytes, but in some conditions neurons also can express it [165]. Although tenascin C is an extracellular matrix (ECM) protein, it might be used as a target to dock exosomes and favor its endocytosis into cells of the niche. In fact, a similar phenomenon occurs with other ECM components such as heparan sulfate proteoglycans (HSPGs) that function as internalizing receptors of cancer cell-derived exosomes [115]. A mimetic peptide that specifically binds tenascin C has already been developed [166].

There is a paucity of information about enriched proteins in cells from other neurogenic niches such as SGZ and spinal cord. Development of transgenic animals, cell sorting [167, 168], and laser capture microdissection [169] of NSCs from the adult brain might help to fill this gap.

Several proteins that mediate neurogenic niche maintenance and precursor differentiation such as Notch, EGFR [170], and Noggin/BMP [171] have been identified. This is a passionate field in neuroscience and there is a high volume of complex data trying to define the key steps on maintenance of NSCs and differentiation of NPCs into specific CNS cell types [172–174]. Thus, the right pathway to modify in the neurogenic niche for a therapeutic purpose is highly speculative. However, as we have already mentioned, the miRNA content of exosomes seems to be the most effective molecular cargo to induce cellular responses potentially appropriate for therapy.

### 8.1. Engineering Exosomal Cargo: What miRNAs Should Be Transferred by Exosomes in Order to Induce Neurogenesis/Gliogenesis and Achieve Recovery on CNS Pathologies?

A number of miRNAs have been shown to be important in the regulation of neurogenesis [175] and gliogenesis [176]. In the neurogenic niche, there is a continuous production of NPCs that commit to a glial or neuronal fate by a series of complex molecular mechanisms [177]. Among the NPC molecules mediating this process, Notch is considered to be a master regulator of neural stem cells and neuronal development [178–181]. In general, its activation is related to maintenance of stemness in the niche and inhibition of neurogenesis [182]. Notch receptors and ligands (e.g., Delta1) are both transmembrane proteins and thus suitable to develop an exosome targeted therapy. A well studied downstream target of Notch that has been shown to inhibit neurogenesis is the transcription factor hairy and enhancer of split 1 (Hes1) and it is known that traumatic brain injury induces Hes1 downregulation as a way to increase neurogenesis and adapt to damage. In fact, downregulation of Hes1 via stereotaxic injection of RNA interference (RNAi) into the hippocampus (targeting SGZ) of rodents results in a significant increase in neuronal production and promotes the differentiation of NPCs into mature neurons in the DG, thus improving cognitive abilities after traumatic brain injury [181]. Interestingly, several miRNAs have been identified to target Notch and its signaling related elements like Hes1; among them, miR-9 is one of the most studied elements for neurogenesis [183]. miR-9 as well as miR-124 directly induces reduction of Hes1 levels and, in general, promotes neuronal differentiation [184]. miR-124 is also upregulated in SVZ after stroke, suggesting a role in functional recovery [185]. In relation to the miRNAs that promote glial fate and generation of astrocytes or oligodendrocytes from NPCs, expression of let-7 has been shown to promote glial fate and its inhibition produces neuronal commitment [186].

Another relevant miRNA target regulating NPCs differentiation (also in SVZ) is SIRT1, a protein deacetylase implicated in energy metabolism. Inhibition of this protein increases the production of new oligodendrocyte precursor cells (OPCs) in the brain and attenuates symptoms in mouse models of demyelinating injuries [75]. Thus, SIRT1 regulating miRNAs such as miR-204-5p [187] might be useful to load SVZ targeted exosomes and potentially treat MS.

It has been recently demonstrated that exogenous miRNAs can be incorporated into exosomes of MSCs and be functionally delivered to neural progenitor cells and astrocytes, modifying the expression of several genes in recipient cells [188]. Thus, as postulated in the review, given the capacity to target a specific cell type by engineering the appropriate surface proteins in exosomes, engineered MSC-derived exosomes may provide an efficient route of therapeutic miRNA delivery to certain cellular components of the neurogenic niche in particular pathological conditions. More research and a deeper understanding of the exosomal surface proteins necessary to target a specific cell type are needed to test this assumption.

**Figure 2:**
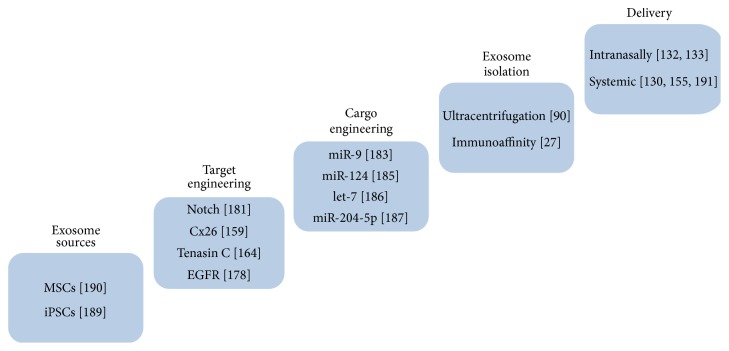
Schematic flowchart to develop a therapy based on the selective delivery of miRNAs mediated by engineered exosomes targeting neurogenic niches.* Exosome sources* box shows the cell culture type in which exosomes will be produced and harvested;* target engineering* box shows neurogenic niche-associated proteins that could be targeted with exosomes engineered to harbor a mimetic binding peptide in the outer surface of the vesicle;* cargo engineering* box shows several miRNAs that are known to modulate the fate of NPCs in the neurogenic niche;* exosome isolation* box shows two strategies to isolate the engineered exosomes from culture media: ultracentrifugation or immunoprecipitation of vesicles using antibodies against the extracellular domain of an exosome marker protein;* delivery* box shows two noninvasive CNS interventions: intranasal and systemic (by bloodstream) incorporation of engineered exosomes. References: iPSCs [189]; MSCs [190]; Cx26 [159]; Tenascin C [164]; EGFR [178]; Notch [181]; miR-9 [183]; miR-124 [185]; miR-204-5p [187] let-7 [186]; ultracentrifugation [90]; immunoaffinity [27]; intranasally [132, 133]; systemic [130, 155, 191].

With all this information, we propose in Figure 2 a schematic flowchart to develop a therapy based on the selective delivery of miRNAs mediated by engineered exosomes to target the neurogenic niche. MiRNA loading can be achieved by transfecting the exosome producing cells with an overexpression plasmid [89] or even by direct electroporation of the mature miRNA into the vesicles [129].

## 9. Exosomes as Diagnostic Tools of CNS Diseases

It is well known that different kind of cells produces exosomes with a specific parental molecular signature [192]. For example, B cell receptor is selectively expressed on B cell-derived exosomes, as CD11c, a specific marker of dendritic cells (DC), is present on DC-derived exosomes [106]. Similarly, oligodendrocyte derived exosomes contain the myelin associated proteins PLP/DM20 [193]. Coherently, exosome cargo depends on the physiological/pathophysiological state of the cell that produces it [194]; for instance, inflammatory and hypoxic stimuli change the protein and RNA content of endothelial cell-derived exosomes [195].

In the CNS, cultured cells of the highly malignant brain tumor glioblastoma multiforme grown under hypoxic conditions secrete exosomes enriched in hypoxia-regulated mRNAs and proteins like caveolin 1, which is also increased in exosomes isolated from the plasma of glioblastoma patients with a poor prognosis [196]. Similarly, it was found that glioblastoma-specific epidermal growth factor receptor vIII (EGFRvIII) is enriched in exosomes isolated from the serum of glioblastoma patients [197, 198]. In fact, exosomes contain each of the toxic protein types associated with neurodegenerative disorders such as HD, PD, AD, and prion disease [199]. Moreover, tau phosphorylated at Thr-181, a biomarker for AD, is elevated in exosomes isolated from the cerebrospinal fluid from AD patients [200].

Thus, exosomes have a great potential as noninvasive diagnostic tools under several CNS pathological conditions. Considering that several pathologies are related to alterations of the stem cell niche, it is intriguing to know whether changes in the molecular signature of specific stem niche derived exosomes are induced in response to neuropathological conditions. A good challenge to solve this question is to identify a specific transmembrane protein that might be used as a marker to capture peripherally the exosomes derived from CNS stem cell niches. Thus, with these exosomes, it would be possible to study the physiological state of a niche.

## 10. Conclusion

The challenges faced when trying to improve the conditions of those affected by neurological disorders have urged researchers to explore new methodologies and to think “outside of the box.” The use of stem cell transplants for therapeutic use is a growing field that, though in its infancy, is showing promising potential to treat neurodegenerative disorders, though their potential risks (e.g., allogenic immune response and the potential of tumor formation) have hindered progress in this field. Interest has therefore shifted for many researchers towards the exosomes liberated by these cells. Besides the compelling possibility of using them as biomarkers, these nanovesicles present a number of advantages that make them uniquely suited as therapeutic agents. (1) They are able to cross the blood-brain barrier; thus, they can be delivered through minimally invasive methods (e.g., blood and/or intranasal delivery). (2) Their content can be manipulated to suit specific needs. (3) The membrane proteins expressed by exosomes could be engineered to target them to precise cell types, improving the specificity of a treatment and thus reducing the incidence of side effects. (4) The possibility of targeting exosomes to specific cell types would open up the door for treatments targeted at the adult neurogenic niches of the brain. These areas of the brain have been insofar unexplored as sites for potential treatment, but their close association to a wide number of different neurological disorders makes their modulation worth considering when seeking for novel therapeutic methods.

Stem cells and the exosomes released by them thus open a vast array of new options for the treatment of disabling neurological pathologies that, due to the complexity of the brain and the difficulty of accessing some of its areas, still remain largely incurable despite decades of intense research.
